# Transcatheter device closure of post–myocardial infarction ventricular septal rupture: A single-centre experience

**DOI:** 10.1016/j.ihj.2026.04.001

**Published:** 2026-04-02

**Authors:** Praveen Chandra, Nagendra Singh Chouhan, Seenu Prasanth Adimoulame, Siddarth Varshney, Deepti Yadav, Anushree Kumbhalkar

**Affiliations:** Heart Institute, Medanta – The Medicity, Sector 38, Gurugram, Haryana, 122001, India

**Keywords:** Post-myocardial infarction ventricular septal rupture, Transcatheter device closure, Cardiogenic shock

## Abstract

Post–myocardial infarction ventricular septal rupture is a rare but fatal mechanical complication, frequently complicated by cardiogenic shock. We conducted a single-centre retrospective cohort study of seventeen surgically declined patients who underwent transcatheter device closure between 2014 and 2025. Cardiogenic shock was present in 15 of 17 patients (88.2%). Device implantation achieved high technical success; however, early mortality remained substantial, with only six patients (35.3%) surviving beyond 30 days. Deaths were mainly due to refractory shock and multiorgan failure. Survival was associated with lower SCAI shock stage, preserved lactate clearance, and higher arterial pH, underscoring the importance of physiological status.

## 1 Introduction

Ventricular septal rupture (VSR) is an uncommon but often fatal mechanical complication of acute myocardial infarction, particularly in patients presenting with cardiogenic shock.^1^^,^^2^ Post-infarction VSR represents a severe haemodynamic insult characterised by acute left-to-right shunting, reduced effective cardiac output, pulmonary overcirculation, right ventricular overload, and systemic end-organ hypoperfusion.^3^ Surgical repair remains definitive therapy; however, operative mortality is high in unstable patients, and delay for tissue stabilisation is often precluded by progressive haemodynamic deterioration.^4^ Transcatheter device closure has emerged as an alternative or salvage strategy in selected high-risk individuals.^5^ Uncertainty persists regarding optimal timing and patient selection. Contemporary Indian data on percutaneous closure remain limited.^6^ We report our institutional experience with transcatheter closure of post-myocardial infarction VSR.

## 2 Methods

This single-centre retrospective observational cohort study included all consecutive surgically declined patients who underwent transcatheter device closure for post–myocardial infarction VSR between 2014 and 2025 at a tertiary centre. The Institutional Ethics Committee approved the study with waiver of consent.

Clinical data included demographics, risk factors, infarct characteristics, VSR timing, and hemodynamic status. Cardiogenic shock was defined as systolic blood pressure <90 mmHg (or requirement of vasoactive support to maintain ≥90 mmHg) for ≥30 min, accompanied by clinical or biochemical evidence of end-organ hypoperfusion. Use of inotropes, ventilation, renal replacement therapy, IABP or other mechanical circulatory support (MCS) was recorded.

Transthoracic and transesophageal echocardiography assessed VSR location, size, shunt, ventricular function, pulmonary pressures, and valves. Procedural variables included access, device type/size, and outcomes. Device selection was based on defect morphology and operator judgment. Coronary angiography was performed when feasible. Timing and circulatory support were determined by the heart team. Laboratory parameters (including MELD-XI from bilirubin/creatinine [mg/dL] and arterial pH) were recorded. Lactate clearance was calculated as [(initial lactate − follow-up)/initial] × 100 within 24 h of closure. Procedural success was defined as device deployment without embolisation or emergency surgical conversion. The primary outcome was survival beyond 30 days. Data were analysed using SPSS v29.0. No missing data were present. Comparisons were performed using Fisher's exact and Mann–Whitney *U* tests. Findings are presented as exploratory associations given the sample size.

## 3 Results

Seventeen patients underwent transcatheter closure for post–myocardial infarction VSR. Mean age was 68 ± 9 years; 11 (64.7%) were male. Diabetes and/or hypertension were present in 10 (58.8%). Fifteen patients (88.2%) were transferred following the diagnosis of VSR. Echocardiography demonstrated apical or anterior mid-septal defects with left-to-right shunting in 16 (94.1%), predominantly following anterior MI with LAD culprit lesions. One patient with inferior MI (RCA culprit) had a basal mid-septal defect. Culprit-vessel PCI was performed in 11 (64.7%), mainly at referring centres. One patient developed VSR after thrombolytic therapy.

VSR was diagnosed a median of 3 days after MI (IQR 1-7), and closure was performed a mean of 5.5 ± 8.0 days later. One patient underwent closure for a residual post-surgical defect. SCAI shock stage distribution differed significantly between survivors and non-survivors, with most survivors presenting in stages B/C (83.3%) and all non-survivors in advanced stages D/E (100%).

Closure was performed via femoral arterial and jugular venous access. Defects were crossed using a hydrophilic guidewire, followed by creation of an arteriovenous loop. Muscular septal occluder devices were deployed, typically oversized by 4–6 mm. Devices used were Amplatzer Muscular VSD Occluders (12, 70.6%), Amplatzer P.I. Muscular VSD Occluders (3, 17.6%), Amplatzer Septal Occluder (1, 5.9%), and a Lifetech ASD device (1, 5.9%). ASD devices were selected in two cases based on anatomical suitability and device availability at the time of intervention. Device position and residual shunting were confirmed by angiography and echocardiography.

**Fig. 1:**
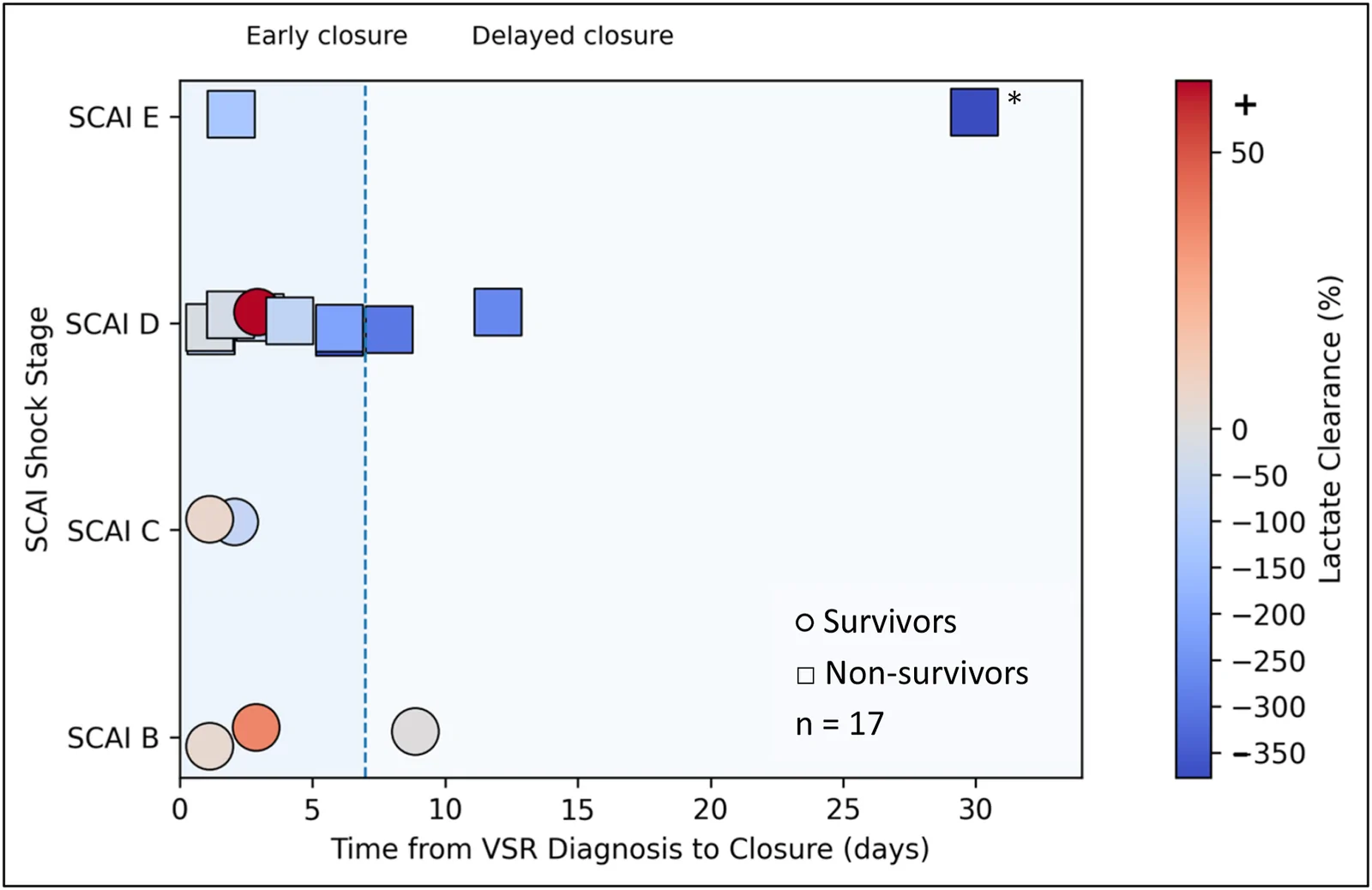
**Timing of transcatheter VSR closure relative to shock severity and lactate clearance.** Each marker represents an individual patient plotted by time from VSR diagnosis to device closure (days) and SCAI shock stage at presentation. Marker colour indicates lactate clearance (%) within 24 h of device closure. Circles denote survivors and squares denote non-survivors. The dashed vertical line represents the cohort median time to closure. ∗ Delayed closure at 30 days was performed for residual VSR following prior surgical patch repair.

Procedural success was achieved in 15 patients (88.2%). One patient developed right ventricular free wall rupture with tamponade, resulting in intraprocedural mortality (supplementary video 1). Another with a basal mid-septal defect had moderate residual shunting (supplementary video 2). No device embolisation, stroke, or emergency surgical conversion occurred. Most patients required inotropic support; IABP was used in 14 (82.4%). No advanced percutaneous LV MCS were utilised. Non-survivors demonstrated impaired lactate clearance (Table 1). In-hospital mortality was 11 (64.7%), attributed to cardiogenic shock alone (7, 63.6%) or with sepsis (4, 36.4%). Death occurred a median of 2 days after closure. Six patients (35.3%) survived beyond 30 days. Fig. 1 illustrates timelines; Table 1 summarises comparisons.

**Table 1:** Exploratory comparison of survivors and non-survivors after transcatheter closure of post–myocardial infarction ventricular septal rupture.

Variable	Survivors (*n* = 6)	Non-survivors (*n* = 11)	*p* value^a^
Clinical characteristics			
Any comorbidity (DM or HTN), n (%)	3 (50.0)	7 (63.6)	0.64
Single-vessel disease (SVD), n (%)	3 (50.0)	7 (63.6)	0.64
Culprit vessel – LAD, n (%)	5 (83.3)	11 (100)	0.35
Culprit vessel – RCA, n (%)	1 (16.7)	0 (0)	0.35
VSR size (mm)^b^	11.5 (9.3–13.8)	12.0 (12.0–16.5)	0.31
Timing from Myocardial Infarction to VSR closure	8.0 (5.0-10.2)	5.0 (4.0-10.0)	0.84
Hemodynamic status and organ support			
Advanced SCAI shock stage (D/E), n (%)	1 (16.7)	11 (100)	**<0.001**
Mechanical ventilation, n (%)	2 (33.3)	9 (81.8)	0.11
Renal replacement therapy, n (%)	0 (0)	5 (45.5)	0.10
IABP use, n (%)	4 (66.7)	10 (90.9)	0.51
RV dysfunction, n (%)	3 (50.0)	7 (63.6)	0.64
PASP >50 mmHg, n (%)	3 (50.0)	5 (45.5)	1.00
LVEF (%)^b^	32.5 (30.0–35.0)	30.0 (27.5–35.0)	0.72
Laboratory and metabolic parameters			
MELD-XI score^c^	17.9 (13.8–21.3)	24.7 (21.8–30.4)	**0.01**
Lactate clearance (%)^b^	−0.1 (−4.7 to 27.8)	−124.8 (−285.5 to −72.2)	**<0.001**
Arterial pH^c^	7.437 (7.351-7.50)	7.104 (7.015-7.164)	**0.001**
CRP peak (mg/L)^b^	48.8 (24.7-69.4)	80.0 (58.0-118.0)	0.122
NT-proBNP (pg/mL)^b^	23,150 (7650–39,400)	30,500 (12,350–46,500)	0.462
Troponin peak (ng/mL)^b^	1.93 (1.37-2.0)	47.4 (24.68-58.35)	**0.003**
Procalcitonin (ng/mL)^b^	0.48 (0.19-1.12)	4.9 (2.85-15.7)	**0.007**
Serum albumin (g/dL)^b^	3.4 (3.0–3.7)	3.0 (2.2–3.3)	0.12

RV – Right ventricle, PASP – Pulmonary artery systolic pressure, LVEF – Left ventricle Ejection Fraction.

a Fisher's exact test for categorical variables; Mann–Whitney *U* test for continuous variables (exploratory).

b Median (interquartile range) for complete data (no missing values).

c MELD-XI is a dimensionless score derived from serum bilirubin and creatinine (mg/dL); arterial pH is unitless.

## 4 Discussion

This single-centre retrospective cohort highlights the persistent lethality of post–myocardial infarction VSR, with high mortality despite satisfactory procedural success; only six patients (35.3%) survived beyond 30 days. Contemporary series report similar early mortality, particularly in those presenting with cardiogenic shock.^1^, ^2^, ^3^^,^^7^ These findings suggest defect closure alone may be insufficient once advanced systemic derangement is established.

Post-infarction VSR represents an evolving haemodynamic catastrophe. Acute left-to-right shunting reduces effective cardiac output, leading to pulmonary overcirculation and systemic hypoperfusion.^2^^,^^3^ Outcomes were associated with shock severity at presentation, with advanced SCAI stage strongly associated with mortality, indicating that mechanical correction may not reverse established metabolic collapse.^2^^,^^8^

Metabolic indices were associated with outcomes. Non-survivors had impaired lactate clearance, lower arterial pH, and higher MELD-XI scores, consistent with severe hypoperfusion; higher troponin likely reflected greater infarct burden. Recovery appeared more dependent on physiological reserve than defect characteristics.^3^^,^^5^ VSR location and timing of closure were not associated with survival.^7^^,^^9^ Closure after advanced metabolic acidosis and refractory hypoperfusion may offer limited benefit, whereas intervention during reversible shock may improve outcomes.^3^

Several practical lessons emerge. Early VSR recognition, rapid transfer, and structured assessment of shock and metabolic status are critical. Early MCS may stabilise selected patients; strategies should account for shunt physiology and ventricular loading. Although guidelines do not support routine IABP use in cardiogenic shock, IABP offers modest haemodynamic support and was used selectively. Contemporary management emphasises physiology-guided stabilisation and tailored MCS approaches.^10^ Anticipatory planning is essential in anatomically challenging defects with limited landing zones.

This study is limited by its retrospective design and small sample. In conclusion, transcatheter VSR closure is feasible, but survival depends on shock severity and metabolic failure. Improved outcomes likely depend on earlier diagnosis, timely MCS, physiology-guided stabilisation, and intervention before irreversible organ injury.^3^

## Declaration of generative AI and AI-assisted technologies in the manuscript preparation process

During the preparation of this work, the authors used **ChatGPT (OpenAI, GPT-5.2)** to assist with language refinement and editorial structuring. The authors critically reviewed, revised, and approved all content and take full responsibility for the accuracy and integrity of the final manuscript.

## Declaration of competing interest

The authors declare that they have no known competing financial interests or personal relationships that could have appeared to influence the work reported in this paper.
